# Left Ventricular Remodeling Following Balloon Mitral Valvuloplasty in Rheumatic Mitral Stenosis: Magnetic Resonance Imaging Study

**DOI:** 10.3389/fcvm.2021.674435

**Published:** 2021-06-04

**Authors:** Amir Anwar Samaan, Karim Said, Wafaa El Aroussy, Mohammed Hassan, Soha Romeih, Amr El Sawy, Mohammed Eid Fawzy, Magdi Yacoub

**Affiliations:** ^1^Faculty of Medicine, Kasr Al Ainy Hospital, Cairo University, Cairo, Egypt; ^2^Magdi Yacoub Heart Foundation-Aswan Heart Centre, Cairo, Egypt; ^3^Department of Cardiothoracic Surgery, Imperial College London, London, United Kingdom

**Keywords:** mitral stenosis, balloon mitral valvuloplasty, myocardial tagging, left ventricular function, left ventricular remodeling, left ventricular deformation, cardiac magnetic resonance imaging

## Abstract

**Background:** Rheumatic heart disease affects primarily cardiac valves, it could involve the myocardium either primarily or secondary to heart valve affection. The influence of balloon mitral valvuloplasty (BMV) on left ventricular function has not been sufficiently studied.

**Aim:** To determine the influence of balloon mitral valvuloplasty (BMV) on both global and regional left ventricular (LV) function.

**Methods:** Thirty patients with isolated rheumatic mitral stenosis (MS) were studied. All patients had cardiac magnetic resonance imaging (CMR) before, 6 months and 1 year after successful BMV. LV volumes, ejection fraction (EF), regional and global LV deformation, and LV late gadolinium enhancement were evaluated.

**Results:** At baseline, patients had median EF of 57 (range: 45–69) %, LVEDVI of 74 (44–111) ml/m^2^ and LVESVI of 31 (14–57) ml/m^2^ with absence of late gadolinium enhancement in all myocardial segments. Six months following BMV, there was a significant increase in LV peak systolic global longitudinal strain (GLS) (−16.4 vs. −13.8, *p* < 0.001) and global circumferential strain (GCS) (−17.8 vs. −15.6, *p* = 0.002). At 1 year, there was a trend towards decrease in LVESVI (29 ml/m^2^, *p* = 0.079) with a significant increase in LV EF (62%, *p* < 0.001). A further significant increase, compared to 6 months follow up studies, was noticed in GLS (−17.9 vs. −16.4, *p* = 0.008) and GCS (−19.4 vs. −17.8 *p* = 0.03).

**Conclusions:** Successful BMV is associated with improvement in global and regional LV systolic strain which continues for up to 1 year after the procedure.

## Introduction

Although, the incidence of rheumatic fever and its complications has declined in developed countries, the disease is still a major health problem in many developing countries (1). It is estimated that up to 30 million schoolchildren and young adults have chronic rheumatic heart disease worldwide, and nearly a third of these have mitral stenosis (MS) (2).

The procedure of balloon mitral valvuloplasty (BMV) has been first described in 1984 by a Japanese cardiac surgeon called Kanji Inoue (3). The main mechanism of successful BMV relies on splitting of the fused commissures and in comparison to surgical commissurotomy it has comparable success rates with better long-term outcomes (4, 5).

Impaired left ventricular (LV) systolic function has been reported in around 30% of patients with MS (6), while in some recent studies, underlying abnormal LV contractility has been described using tissue Doppler Imaging (TDI) and Speckle Tracking Echocardiography (STE) in MS patients with apparently normal LV systolic function (7, 8).

Changes taking place in LV following BMV have been a subject of investigation and debate over the past years. Different diagnostic tools including cardiac catheterization and angiocardiography (9), echocardiography (10), TDI (11), and STE (12) were used earlier for that purpose. Whereas, some of these studies showed improved LV function after BMV (13, 14) others failed to show any change following the procedure (15, 16).

## Purpose of the Study

The present study aimed to determine the impact of BMV on LV volumes, global, and regional function using CMR, 6 months and 1 year following BMV in patients with isolated rheumatic MS.

## Methods

### The Study Population

The study was an observational prospective cohort study that took place at a tertiary referral hospital. The study included 30 consecutive patients with isolated rheumatic MS who underwent successful BMV, and a control group of 12 healthy volunteers without known prior cardiovascular disease.

BMV was considered indicated for symptomatic patients with severe MS (mitral valve area (MVA) < 1.5 cm^2^) and favourable valve morphology (Wilkin's score of 11 or less and the absence of commissural calcifications) in the absence of left atrial thrombus or moderate-to-severe mitral regurgitation (17). A successful BMV was defined as an immediate final MVA of more than 1.5 cm^2^ or a 40% increase in MVA with no mitral incompetence beyond mild severity and with no procedure-related complications (18, 19). Exclusion criteria for this study included conditions that may alter LV function e.g., coronary artery disease, uncontrolled systemic hypertension, more than mild aortic valve disease, or any advanced systemic disease as well as patients with contraindications to CMR e.g., claustrophobia or metallic implants. The study was conducted after the approval of institutional Research Ethics Committee of Aswan Heart Centre, Aswan, Egypt, with complete adherence to all required institutional safety measure. The study protocol was illustrated to all the patients and a written informed consent was obtained from all the participants before being enrolled in the study.

Patients referred to the hospital were first screened for eligibility for this study. Eligible patients had baseline workup that included clinical evaluation, electrocardiogram (ECG), echocardiography study [transthoracic and trans-oesophageal (TEE)], CMR imaging and invasive haemodynamic study before BMV. Only patients who had a successful procedure were recruited and then had two follow up studies at 6 months and 1 year after the procedure. Medical treatment, in the form of beta-blockers and a small dose of diuretics, was initiated at least 6 months pre-procedural and was kept unchanged throughout the follow-up period. On the other side, individuals in the control group were evaluated clinically and had ECG and trans-thoracic echocardiography to exclude any cardiovascular disease and then had a complete CMR imaging evaluation.

### Echocardiography Study

Transthoracic echocardiography images were obtained *via* Philips iE33 with 2.5 MHz sector transducer. All studies were done according to the criteria provided by the American Society of Echocardiography (ASE) (20). LV end-systolic and end-diastolic volumes, EF, left atrial volume, MVA (planimetry), mean pressure gradients, Wilkin's score and degree of mitral regurgitation were estimated. TEE was performed for all the patients to exclude left atrial appendage thrombus before valvuloplasty.

### Invasive Haemodynamic Measurements

Haemodynamic assessment was performed *via* right and left heart catheterization immediately before BMV. Tracings were obtained using AXON Sensis XP (Siemens Healthcare Systems) using a standardized protocol under stable conditions. Left atrial pressure (LAP), mean pressure gradient, systemic vascular resistance (SVR), pulmonary artery pressure (PAP), and cardiac index were measured.

### Balloon Mitral Valvuloplasty

All patients had percutaneous BMV by antegrade trans-septal approach with the use of the Inoue balloon catheter (Toray Medical Co., Tokyo, Japan) using a standardized technique (3). Immediate post-procedural haemodynamic assessment of trans-mitral pressure gradients and left atrial pressure was performed.

### Cardiac Magnetic Resonance Imaging

CMR examination was performed using 1.5 Tesla Siemens Aera (Siemens Medical System, Erlangen, Germany), with 25-m T/M maximum gradient strength and a phased array cardiac coil of 48 channels.

#### Image Acquisition

Systolic and diastolic volumes were assessed using a retrospective ECG-gated steady-state free precession (SSFP) sequence during breath-holding. Vertical long-axis 2- and 4- chamber views and short-axis views consisting of 12–14 contiguous slices were acquired, covering both ventricles from the base of the heart to the apex.

For the tissue characterization (viability) studies, a bolus of 0.2 mmol/kg of Gadolinium was infused at an injection rate of 4 ml/s followed by a bolus of 20 ml of normal saline, infused at the same rate. Delayed enhancement 3D acquisitions were acquired in diastole 10 min after Gadolinium injection with a time of inversion (TI) of 175–300 ms. Tissue characterization studies were performed only once for MS patients at baseline.

#### Myocardial Tagging

Tagging MR images were performed in the short heart axis orientation and then in apical four-chamber, apical two-chamber and apical long-axis planes. Three short-axis planes were obtained (basal, mid and apical). A segmented two dimensional electrocardiographically triggered fast low angle shot pulse sequence (field of view 240 × 320 mm^2^, matrix 216 × 256, TR/TE 9.0/4.0, flip angle 15°) was used in the cine mode. A rectangular grid with a spacing of 8 mm was applied. The acquisition window for one cardiac phase was 70–90 ms. resulting in a temporal resolution of 35–45 ms.

### Image Analysis

**Figure 1:**
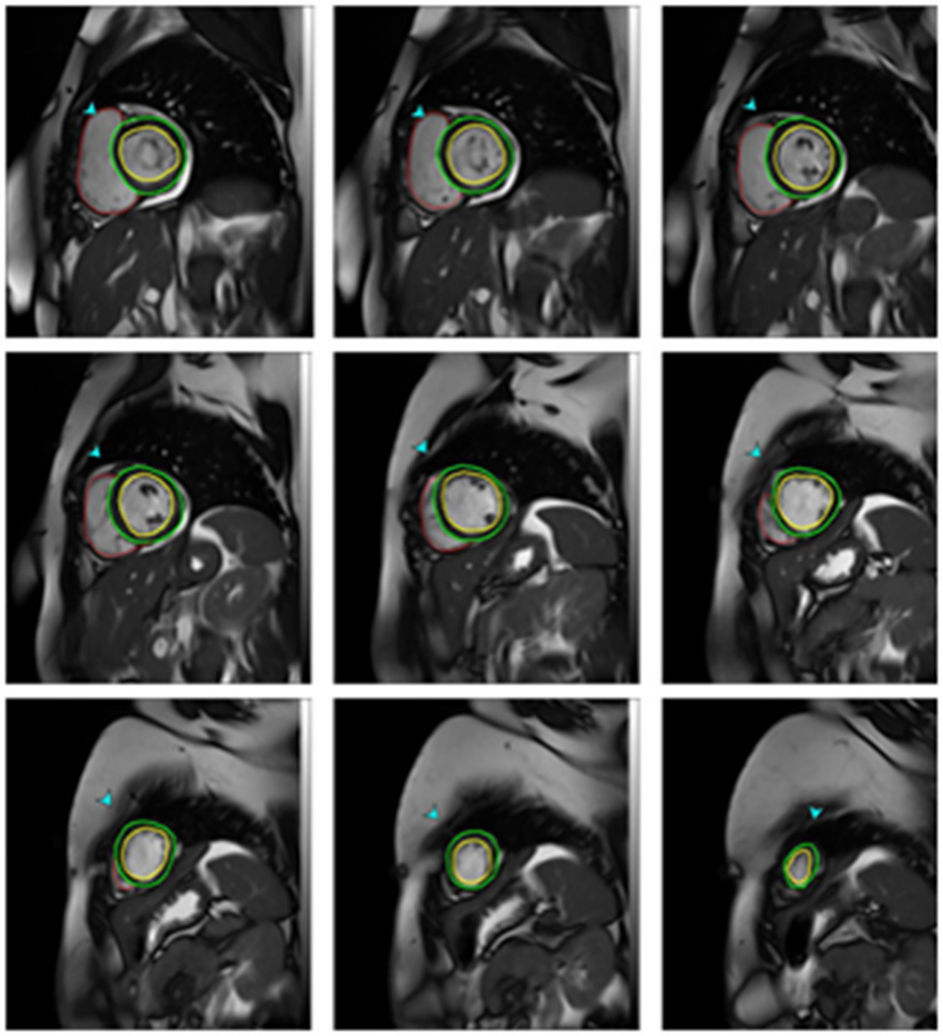
CMR Measurements of ventricular volumes in one of mitral stenosis patients. End diastolic short-axis images from the apex to the base with epicardial (green lines) and endocardial (red lines) contours drawn for the left ventricle and endocardial contours for the right ventricle.

Ventricular volumes and function were analyzed using the SYNGO software (Siemens health system). After the determination of the end-diastolic and the end-systolic frame on the first basal slice to show circumferential myocardium at both diastole and systole, the endocardial contour was traced manually. All volumes were calculated automatically by summing the areas in the entire series of short-axis cine images (Figure 1). Volumes were then indexed to body surface area (BSA) and LV ejection fraction (EF) was calculated (21).

### Strain Measurement

**Figure 2:**
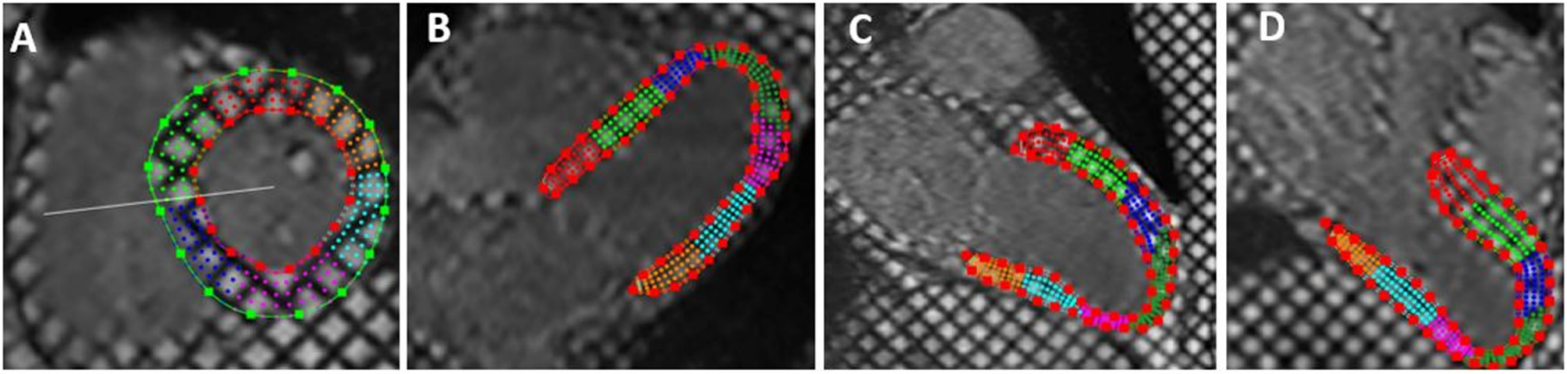
Tagged cardiac magnetic resonance imaging with epicardial and endocardial border tracing in: short axis **(A)**, horizontal long axis (apical four chambers) **(B)**, vertical long axis (apical two chambers) **(C)**, and left ventricular outflow view (three chambers views) **(D)**. The short axis images were used for estimation of circumferential strain while long axis views were used for estimation of longitudinal strain.

**Figure 3:**
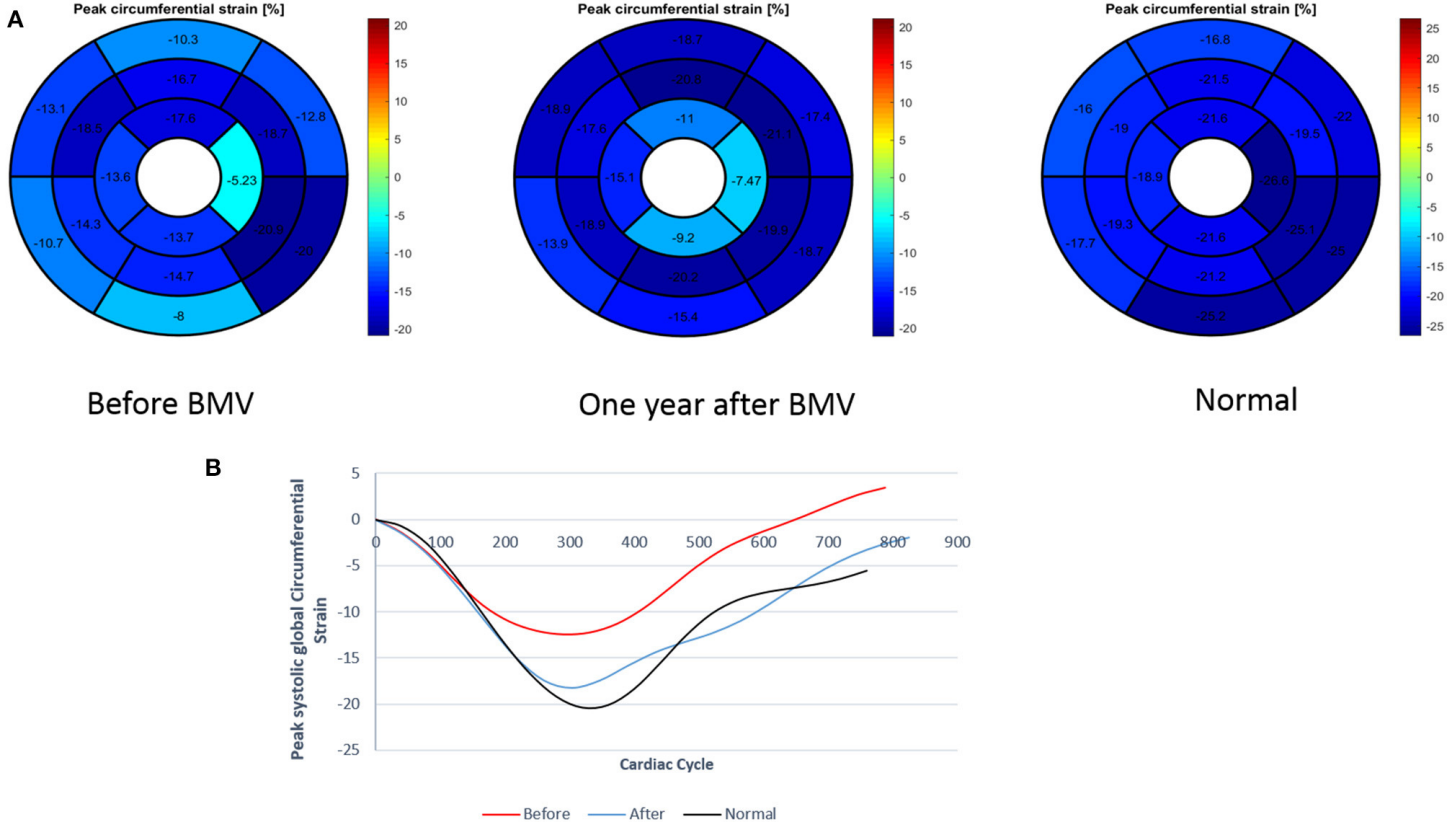
**(A)** Measurement of regional and global circumferential strain (Bull's eye plot) in a patient with mitral stenosis before and 1 year after BMV with comparison to a control individual **(B)** Graph representation of GCS in a patient with mitral stenosis before and after BMV in comparison to a control individual.

Tagged MRI images were analyzed quantitatively using the software HARP (Harmonic Phase Imaging, version 5, Diagnosoft). After adjusting the Region of Interest (ROI) and manual defining of the endocardial and epicardial borders, myocardial strain curves throughout the cardiac cycle were automatically generated for the left ventricular 17 segments. Peak systolic longitudinal strain was measured for different myocardial segments from the tagged apical 2, 3, and 4 chambers views while peak systolic circumferential strain was estimated from tagged short axis views. Strain was measured as the change in myocardial segment length relative to its end-diastolic length. Global strain values were calculated as the mean value of strain measurements of the LV segments. Negative strain values denoted myocardial shortening (22, 23) (Figures 2, 3).

## Statistical Analysis

Statistical analysis was performed using Statistical Package for Social Sciences, version 16 (SPSS 16). Firstly, all variables were tested for normality using Kolmogrov-Smirnov test; If the test was significant, non-normality was accepted otherwise double-check using graphs, skewness and kurtosis were required to confirm normality. All the quantitative variables in this research were not normally distributed and accordingly are presented as median (range). Qualitative data are presented as number (percentage).

Variables were compared between two related samples using Wilcoxon test. Categorical variables were compared using Chi-square analysis. Bivariate correlations were performed using Spearman correlation coefficient. Probability value of < 0.05 was considered statistically significant.

Delta (Δ) for a specific parameter was calculated by subtracting the value of this parameter at follow up from its corresponding value at baseline study.

## Results

Among 78 patients screened for eligibility of BMV, 51 patients had BMV over a 1 year period. Three patients had unsuccessful procedure where two patients had post-procedural severe mitral regurgitation and were referred to surgery, and the procedure failed to attain a satisfactory valve area in the third one. Eighteen patients were excluded due to various reasons including pregnancy, claustrophobia, and refusal to participate in the study. The remaining 30 patients, who had a successful procedure, were included in the study and had two follow up visits at 6 and 12 months after BMV.

The demographics of the study population, clinical characteristics, baseline echocardiographic and heamodynamics data of the patients are summarized in Tables 1, 2.

**Table 1:** Baseline clinical, echocardiographic, and hemodynamic data of the patients.

**Variable**	**Value**
Age (years)	33 (23–41)
Female gender [*n* (%)]	22 (73.3%)
**NYHA**	
II [*n* (%)]	17 (56.6%)
III [*n* (%)]	13 (43.3%)
Atrial fibrillation ryhthm [*n* (%)]	3 (10%)
**Echocardiographic data**	
MVA (cm^2^)	0.9 (0.6–1.3)
Mean pressure gradient (mmHg)	12.5 (8–24)
Wilkin's score	7 (6–10)
PASP (mmHg)	47.5 (25–120)
**Heamodynamics data**	
HR (bpm)	72 (64–88)
LAP (mmHg)	30 (14–35)
LVEDP (mmHg)	12 (8–19)
Mean trans-mitral pressure gradient (mmHg)	14 (9–28)
Mean PAP, (mmHg)	40 (19–72)
CO (L/min)	3.6 (2.5–5.7)
Cardiac index (L/min/m^2^)	2 (1.5–3.1)
SVR (Woods units)	20.4 (14.3–38.2)

*MVA, Mitral valve area; NYHA, New York Heart Association; PASP, pulmonary artery systolic pressure; HR, heart rate; LAP, left atrial pressure; LVEDP, left ventricular end diastolic pressure; mean PAP, mean pulmonary artery pressure; CO, cardiac output; SVR, systemic vascular resistance. Values are expressed as median and range. Data are presented as median (range) or number (%)*.

**Table 2:** Age and gender in both control group and MS patients.

**Parameter**	**Control group** ** (*n* = 12)**	**Baseline MS patients** ** (*n* = 30)**	***P*-value**
Age (years)	31 (21–39)	33 (23–41)	0.81
Female gender, [*n* (%)]	9 (75%)	22 (73.3%)	0.89

### LV Volumes and Function in MS Patients vs. Control Group

Compared to the control group, patients with MS had similar LVEDVI (74 vs. 71 ml/m^2^, *p* = 0.32), significantly larger LVESVI (31 vs. 22 ml/m^2^, *p* = 0.007) and significantly lower LV ejection fraction (57 vs. 64%, *p* = 0.004). MS patients had lower regional peak systolic longitudinal and circumferential strain in all LV myocardial segments comapred to control group. Peak systolic global circumferential strain (GCS) and global longitudinal strain (GLS) were significantly lower in MS patients (−23.2 vs. −15.6%, *p* = <0.001 and −22.7 vs. −13.8%, *p* = <0.001, respectively).

### Tissue Characterization

Late gadolinium enhancement studies showed no evidence of myocardial fibrosis in all MS patients.

### Changes Following Balloon Mitral Valvuloplasty

Following BMV, as evaluated by trans-thoracic echocardiography, there was a significant increase in MVA [1.9 (1.7–2.3) vs. 0.9 (0.6–1.3) cm^2^, *p* < 0.001], a significant drop in mean pressure gradients [5 (2–8) vs. 12.5 (8–24) mmHg, *p* < 0.001] and a significant drop in PASP [35 (20–50) vs. 47.5 (25–120) mmHg, *p* = 0.002].

#### LV Volumes and Ejection Fraction

No significant change was seen in LVEDVI at 6 months and 1 year following BMV. At 1 year, a trend toward a significant decrease in LVESVI was seen (29 vs. 31 ml/m^2^, *p* = 0.079) associated with a significant improvement in LVEF (62 vs. 57%, *p* = 0.002) (Table 3).

**Table 3:** LV volumes, EF, strain and torsion values in control group and in MS patients at baseline, 6 months and 1 year after BMV.

**Parameter**	**Control group**	**Patients at baseline**	**6 months follow-up**	**1 year follow-up**	***P*^**(1)**^**	***P*^**(2)**^**	***P*^**(3)**^**	***P*^**(4)**^**
LVEDVI (ml/m^2^)	71 (64–85)	74 (44–99)	73 (48–117)	73(48–119)	0.32	0.45	0.75	0.65
LVESVI (ml/m^2^)	22 (15–35)	31 (14–57)	30 (20–57)	29 (17–54)	**0.007**	0.14	**0.079**	0.12
LVEF (%)	64 (58–67)	57 (45–69)	58 (50–68)	62 (53–72)	**0.004**	0.244	** <0.001**	**0.002**
LV GLS (%)	−22.7 (−21.1– −24.8)	−13.8 (−10.2– −18.5)	−16.4 (−13.7– −23.1)	−17.8 (−13– −23.9)	** <0.001**	**0.001**	** <0.001**	**0.008**
LV GCS (%)	−23.2 (−22.2– −24.9)	−15.6 (−10.5– −19)	−17.8 (−13.8– −21.9)	−19.4 (−13.6– 23.9)	** <0.001**	**0.002**	** <0.001**	**0.03**

*P^(1)^: Baseline vs. control group, P^(2)^: Baseline vs. 6 months follow up, P^(3)^: baseline vs. 1 year follow up, P^(4)^: 6 months vs. 1 year follow up. LVEDVI, left ventricular end-diastolic volume index; LVESVI, left ventricular end-systolic volume index; EF, ejection fraction; GCS, global circumferential strain; GLS, global longitudinal strain. Bold values refer to statistical significance*.

#### Deformation Analysis

**Figure 4:**
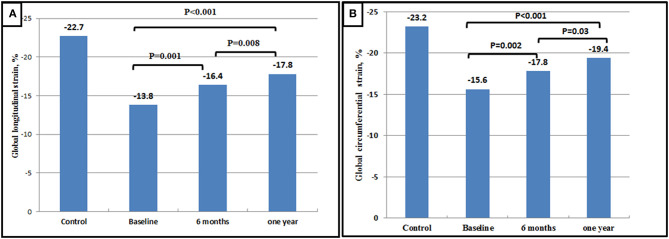
Bar graphs showing Changes in LV GLS after BMV **(A)** and Changes in LV GCS after BMV **(B)**.

**Figure 5:**
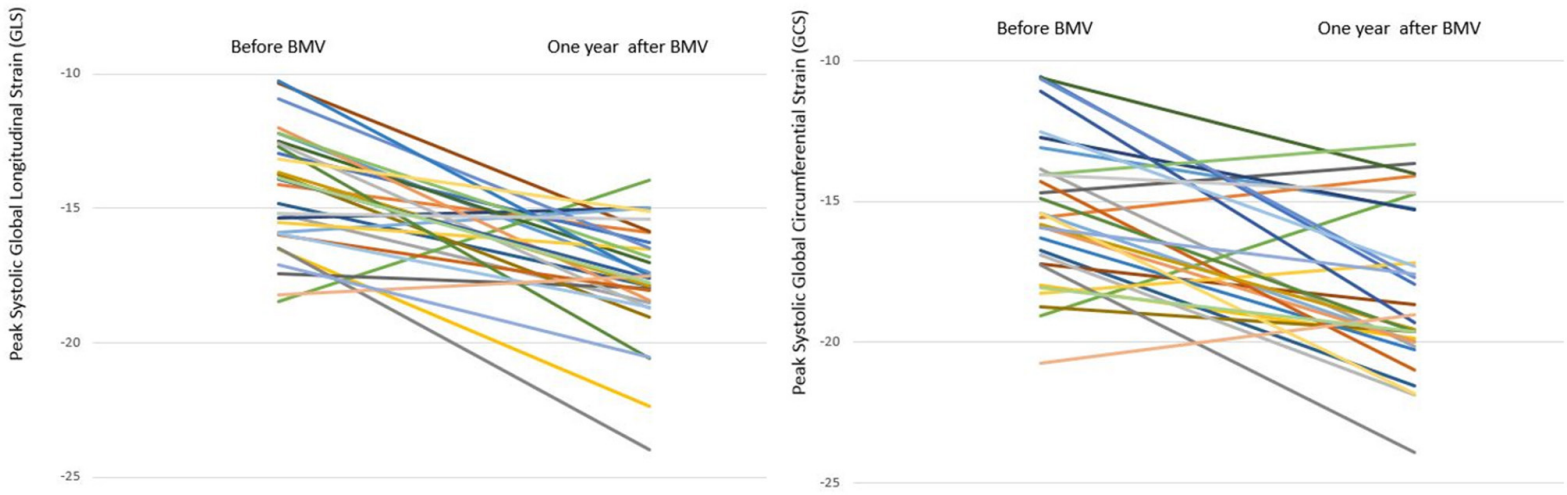
Line chart representing changes in GLS and GCS in individual mitral stenosis patients 1 year after BMV.

- **Regional strain:** At 1 year follow up, an improvement in peak circumferential systolic strain values was noted in all LV myocardial segments; this improvement showed statistical significance in 15 segments and trend toward significance in the remaining two segments. Similarly, all LV myocardial segments showed significant improvement in peak longitudinal systolic strain values at 1 year (Supplementary Tables 1, 2).
- **Global strain:** A significant improvement was shown in LV GLS at 6 months (−16.4 vs. −13.8%, *p* = 0.001) with a further improvement at 1 year (−17.8 vs. −16.4%, *p* = 0.008). Similar changes were observed in GCS with a significant improvement at 6 months (−17.8 vs. −15.6%, *p* = 0.002) and a further improvement at 1 year (−19.4 vs. −17.8%, *p* = 0.03). However, at 1 year following BMV, both GLS and GCS values remained significantly lower than those of the control group (−17.8 vs. −22.7%, *p* < 0.001 and −19.4 vs. −23.2%, *p* < 0.001, respectively) (Figures 4, 5).

**Figure 6:**
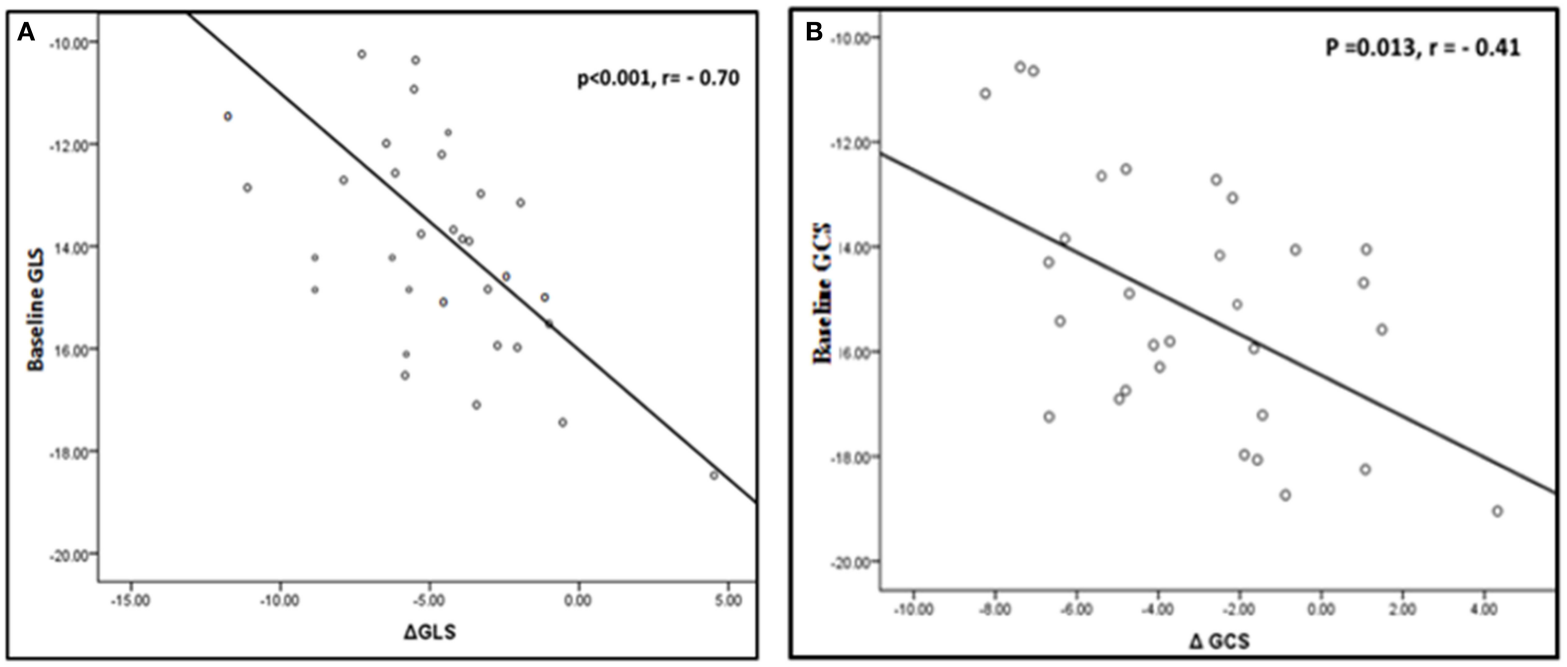
Correlation between change in LV deformation parameters and baseline values. **(A)** Correlation between change in GLS and baseline GLS. **(B)** Correlation between change in GCS and baseline GCS.

Patients with lower pre-procedural left ventricular GLS and GCS experienced significantly higher improvement in 1 year post-procedural strain values (*r* = −0.7, *p* < 0.001 and *r* = −0.4, *p* = 0.013 respectively) (Figure 6). No significant correlation was found between changes in LV deformation parameters and changes in MVA, mean pressure gradient across the mitral valve or LV volumes.

## Discussion

Rheumatic heart disease represents a significant medical challenge in the developing countries. BMV remains the procedure of choice for patients with rheumatic mitral stenosis who have a favorable valve morphology. This type of intervention is still frequently asked for in the developing countries though it is uncommonly needed in the developed countries owing to the remarkably declined incidence and prevalence of RHD in these regions. Surgical intervention, mostly *via* valve replacement, is reserved for patients who have markedly thickened or calcified valves in whom BMV wouldn't be feasible or in the presence of associated significant mitral incompetence.

This study describes the extent, timing and pattern of left ventricular remodeling following BMV. Although, rheumatic heart disease affects primarily heart valves, there is a continuing debate whether the myocardium is affected primarily by the rheumatic process due to molecular mimicry between myosin heavy chain and bacterial proteins or secondary to chronic changes in cardiac output due to valvular disease (24). Furthermore, the influence of relief of the hemodynamic burden of MS on LV function has not been systematically studied. MRI imaging provides an extremely powerful tool to study left ventricular function non-invasively. In addition, including a control group helped define the normal values of myocardial deformation which are known to vary among different racial groups.

### Changes in LV Volumes After BMV

Several previous studies described the change in ventricular volumes following BMV. Mohan et al., using angiocardiography in one study (25) and echocardiography in another one (10), showed no significant change in both LVEDV and ESV following BMV. Both Sengupta et al. (16) and Pamir et al. (15) also showed no significant short term changes in ventricular volumes following BMV. On the contrary, using ventriculography, Goto et al. (13) demonstrated an immediate increase in LVEDVI after successful BMV and recently, Sengupta et al. (26) showed a significant increase in LVEDVI 72 h after BMV in an echocardiography study. Only a few studies evaluated the long term effects of BMV on ventricular volumes. Fawzy et al. (9), using angiography, followed 17 MS patients after BMV and showed a significant increase in LVEDVI immediately after BMV with a further increase at a mean of 12 months follow up. In the present study, using CMR, we demonstrated a decreased LVESVI with no significant change in LVEDVI following BMV.

### Changes in LVEF After BMV

In the present study, as estimated by CMR, a late significant improvement in LVEF was observed at 1 year following BMV.

Using ventriculography, both Mohan et al. (25) and Pamir et al. (15) could not find any significant change in LVEF acutely after BMV. Using echocardiography, Sengupta et al. (16) and Akcakoyun et al. (11) showed no significant acute change in LVEF following BMV. Improved LVEF immediately after BMV was reported by Goto et al. (13), Fawzy et al. (9), and Razzolini et al. (14) using cardiac catheterization and angiography, and by Tischler et al. (27) using echocardiography, and was attributed to improved LV loading conditions. A few studies investigated the long term effect of BMV on LVEF. Following the immediate increase in LVEF after BMV, both Fawzy et al. (9) and Tischler et al. (27) showed a further increase at a mean of 12 and 11 months follow up, respectively.

### Changes in Left Ventricular Deformation After BMV

We demonstrated a significant improvement in both GLS and GCS at 6 months with a further improvement in both parameters at 1 year.

A few previous studies investigated the change in LV deformation following BMV. Sengupta et al. (16), using TDI, showed a significant improvement in mitral annular peak systolic and peak early diastolic excursion 72 h following BMV. Bektaş et al. (28) showed a significant improvement in basal lateral, septal, anterior, and inferior systolic strain 7 days after BMV. Recently, STE was used in two studies for evaluation of changes in LV deformation following BMV. Roushdy et al. showed significant improvement in LV GLS immediately after BMV, a change that was maintained at 3 months follow up (12). Sengupta et al. (26) reported significant improvement in both GLS and GCS 72 h after BMV.

In our study, the improvement in LV deformation after BMV followed by the late improvement in global ejection fraction suggests that myocardial dysfunction in mitral stenosis is reversible and is probably due to long-standing heamodynamic alteration rather than rheumatic inflammatory process. However, at 1 year both GLS and GCS values remained significantly lower than those of the control group. Though, that might be explained by the presence of a residual mild mitral stenosis, it may also suggest an underlying myocardial factor to be contributing to LV abnormalities. Whether the deformation parameters will continue to increase to reach the levels of the control group at a later stage or will remain as impaired as they are, is a question that needs further investigation.

### Suggested Mechanisms of LV Abnormalities and Their Improvement After BMV

#### Loading Conditions

- **After-load:** Increased after-load in MS patients has been described in many previous studies (6, 9, 29). The reduced stroke volume in these patients could result in a compensatory peripheral systemic vasoconstriction leading to increased SVR. To the best of our knowledge, the long term effect of BMV on SVR was investigated in only one previous study where Fawzy et al. (9) showed a significant drop of SVR in MS patients 1 year after BMV.
- **Pre-load & pattern of LV filling:** In previous studies, LVEDV, used as a surrogate for LV pre-load, has been described to be either reduced, normal (29, 30) or even increased (31, 32). Abnormal pre-load might be associated with LV dysfunction in these patients. Recently, with the advent of techniques used for intra-cardiac flow visualization, it was shown that LV systolic function depends not only on the amount of diastolic filling but also on the pattern of filling (33, 34). The flow within the cardiac chambers has been shown to follow a complex sequence characterized by the formation of spiral rings and that pattern is essential to maintain a normal systolic and diastolic performance (34). Sengupta et al. (26, 34) have recently suggested that mitral stenosis might disturb that normal pattern of filling which in turn might contribute to the systolic dysfunction seen in MS patients. Improved pattern of ventricular filling may be one of the factors associated with improved LV function following BMV. This theory, however, requires further investigation.

#### Myocardial Factor

Besides the disturbed loading conditions, it has been debated whether the intrinsic myocardial contractility is normal or impaired in MS patients (6, 29, 35). The myocardial factor as a cause of LV dysfunction in MS patients was best supported in a previous pathological study where ultra-structural pathological changes were observed in myocardial biopsies obtained from MS patients (36). While these pathological alterations were linked by some to a previous rheumatic myocardial process, others related these changes to the chronic abnormalities in ventricular filling (37, 38).

In the present study, the absence of late gadolinium enhancement in all MS patients excludes myocardial fibrosis as a contributing factor of LV dysfunction. On the other hand, it took up to 1 year to see a significant change in LVEF after relieving mitral obstruction and this lag may be the time needed for the aforementioned ultra-structural pathological alterations to reverse after correcting the long-standing abnormal loading conditions.

## Study Limitations

The small number of patients included in the study is one of the main limitations. Another limitation is that the recruited patients represented just a subgroup of MS patients where only patients with favourable valve morphology were included. Patients with heavily affected valves were excluded as well as elderly patients and those with any other valvular affection or comorbidities. In clinical practice, MS patients occasionally present with other conditions and associations that might be interfering with the ventricular recovery observed following BMV in this study.

## Clinical Implications

This study elucidates LV abnormalities associated with rheumatic MS, proves that the associated LV dysfunction is reversible following BMV and clarifies the time frame of LV recovery. In MS patients who present with significantly impaired LV, physicians may hesitate to dilate the valve lest increasing blood flow may have a detrimental effect on LV. However, this study is reassuring that LV undergoes gradual favourable remodeling following BMV and, in addition, patients with lower baseline myocardial strain values appeared to achieve a higher significant improvement at 1 year.

## Conclusions

BMV results in continued slow favourable LV remodeling. This strengthens the recent strategy to make this form of treatment available for the very large number of patients who need it worldwide.

## Data Availability Statement

The raw data supporting the conclusions of this article will be made available by the authors, without undue reservation.

## Ethics Statement

The studies involving human participants were reviewed and approved by Magdi Yacoub Heart Foundation Research Ethical Committee. The patients/participants provided their written informed consent to participate in this study.

## Author Contributions

AS: collecting data and writing the manuscript. KS and SR: conception of the idea, supervising the methodology, and revising the manuscript. WA, MF, and MY: critically revising the manuscript. MH: analysis of the data and statistics. AE: collecting the data. All authors contributed to the article and approved the submitted version.

## Conflict of Interest

The authors declare that the research was conducted in the absence of any commercial or financial relationships that could be construed as a potential conflict of interest.
