# Supplementary material for: Left Ventricular Remodeling Following Balloon Mitral Valvuloplasty in Rheumatic Mitral Stenosis: Magnetic Resonance Imaging Study
Source: Front Cardiovasc Med. 2021 Jun 4;8:674435. doi: 10.3389/fcvm.2021.674435 (PMC8212956; doi:10.3389/fcvm.2021.674435)
Supplement: Supplementary file 1 [file Data_Sheet_1.docx]

**Table 1: Changes in peak systolic circumferential strain in LV myocardial segments following BMV**

| LV segments | Baseline | 6 months follow -up | P ^(1)^ | One year follow-up | P ^(2)^ | P ^(3)^ |
| --- | --- | --- | --- | --- | --- | --- |

| 1.Basal anterior | -12.4 (-4- -19) | -15.4 (-5-19.8) | 0.005 | -16.3 (-11.9- -23.2) | 0.002 | 0.24 |
| --- | --- | --- | --- | --- | --- | --- |
| 2.Basal anteroseptal | -11.6 (-3.2- -19.6) | -15.5 (-4.5- -19.3) | 0.003 | -16.1 (-11.7- -21.2) | 0.001 | 0.04 |
| 3.Basal inferoseptal | -12 (-2.6- -15.7) | -13.5 (-10.5- -20.7) | 0.02 | -15.1 (12.2- -19.6) | 0.02 | 0.90 |
| 4.Basal inferior | -13.5 (-4.3- -22) | -15.6(-16.7- -27) | 0.023 | -18.4 (-11.8- -27) | 0.001 | 0.004 |
| 5.Basal inferolateral | -15.7 (-1.7- -22.8) | -18 (-10.4- -23) | 0.29 | -19.7 (-15.6- -24) | 0.001 | 0.008 |
| 6.Basal anterolateral | -12.4 (-4.2- -21.6) | -15.2 (-6.5- -23) | 0.06 | -18.8 (-13- 27.6) | 0.02 | 0.025 |
| 7.Mid anterior | -17.9 (-10- -20.5) | -19.1 (-14.3- -25) | 0.068 | -20 (14.8- -27) | 0.01 | 0.39 |
| 8.Mid anteroseptal | -14 (-12.1- -19.6) | -16 (-14- -20.1) | 0.01 | -17.3 (13.4- -19) | 0.032 | 0.58 |
| 9.Mid inferoseptal | -13.2 (-10.7- -18.5) | -14.9 (-10.6- -19.7) | 0.03 | -15.6 (12.7- 22.8) | 0.01 | 0.68 |
| 10.Mid inferior | -16 (-4.5- -20.6) | -20 (-9 - -24) | 0.08 | -22 (-11.4- -28.2) | 0.002 | 0.02 |
| 11.Mid inferolateral | -18.3 (-12.1- -21.9) | -20 (-15.2- -29.3) | 0.078 | -22 (-14.2- -26.4) | 0.064 | 0.93 |
| 12.Mid anterolateral | -17.6 (-15.7- -23.3) | -20.9 (-10.9- -25.4) | 0.098 | -23.1 (17.3- -28.4) | 0.009 | 0.050 |
| 13.Apical anterior | -18 (-11.7- -24.3) | -20.1 (-13.4- -24.2) | 0.12 | -21.7 (15.2-24) | 0.13 | 0.36 |
| 14.Apical septal | -14.1 (-9.1- - 19.9) | -16.1 (-10.8- -23.10 | 0.028 | -19.2 (10.4- -25.2) | 0.014 | 0.2 |
| 15.Apical inferior | -15.5 (-5.2- -22.0) | -21 (-7.7- -24.5) | 0.02 | -23 (-9.1- -25) | 0.005 | 0.15 |
| 16.Apical lateral | -20 (-4.2- -27.2) | -21.5 (-12.2- -26.3) | 0.65 | -22.6 (-17.5- -27.8) | 0.009 | 0.04 |

Strain values are defined as percentage shortening (%). P ^(1)^: Baseline vs. 6 months follow up , P ^(2):^ baseline vs. one year follow up, P ^(3)^: 6 months vs. one year follow up. Values are expressed as median and range.

**Table 2: Changes in peak systolic longitudinal strain in LV myocardial segments following BMV**

| Parameter | Baseline | 6 months follow -up | P ^(1)^ | One year follow-up | P ^(2)^ | P ^(3)^ |
| --- | --- | --- | --- | --- | --- | --- |

| 1.Basal anterior | -9.6 (-3- -18) | -11.7 (-6.6- -21.1) | 0.02 | -11.8 (6.4- -29) | 0.009 | 0.03 |
| --- | --- | --- | --- | --- | --- | --- |
| 2.Basal anteroseptal | -10.8 (-7- -16) | -12.7 (-8.4- -17.4) | 0.004 | -14.4 (-8- -22.8) | 0.009 | 0.12 |
| 3.Basal inferoseptal | -13 (-5.4- -16.4) | -15.9 (-10.2- -26.1) | 0.002 | -18.5 (-5.7- -24.2) | 0.005 | 0.2 |
| 4.Basal inferior | -9 (-4.6- -18) | -14 (-5- -24.4) | 0.001 | -16.5 (-7- -24.7) | 0.001 | 0.002 |
| 5.Basal inferolateral | -13.2 (-7.1- -20) | -17.3 (-16- -24.5) | 0.001 | -17 (-5.3- -24.3) | 0.001 | 0.23 |
| 6.Basal anterolateral | -10.8 (-6.7- -17.8) | -12.4 (-9.7- -24) | 0.008 | -16.8 (-12.1- -21.5) | <0.001 | 0.003 |
| 7.Mid anterior | -15.9 (-10.1- -20) | -17.5 (-9.7- -24) | 0.012 | -20 (-11.5- -23.8) | 0.006 | 0.03 |
| 8.Mid anteroseptal | -15.5 (-9- -20) | -17.3 (-11- -22) | 0.03 | -20.5 (-13.5- -29.3) | 0.007 | 0.117 |
| 9.Mid inferoseptal | -14.6 (-3.5- -20.8) | -16.5 (-10.7- -22.4) | 0.015 | -18.5 (-14.3- -26.6) | 0.010 | 0.07 |
| 10.Mid inferior | -12.9 (-5- -18) | -15.3 (-10.7- -23.5) | 0.019 | -18.9 (-7.2- -21.6) | 0.007 | 0.19 |
| 11.Mid inferolateral | -14.5 (-9- -18) | -16 (-8.9- -25.4) | 0.023 | -20.4 (-13.4- -30.3) | 0.006 | 0.02 |
| 12.Mid anterolateral | -12.75 (-4.7- -20.1) | -14.3 (-10.9- -23.2) | 0.01 | -15.9 (-12- - 23.8) | 0.07 | 0.4 |
| 13.Apical anterior | -19 (-15- -22) | -22.7 (--17- -27) | 0.012 | -22.5 (-17.8- -27) | 0.01 | 0.02 |
| 14.Apical septal | -15 (-10- -20.2) | -18.3 (-11.5- -24.6) | 0.11 | -19.4 (-13.9- -29.6) | 0.012 | 0.66 |
| 15.Apical inferior | -14.7 (-3- 18) | -16.9 (-8.1- -23.1) | 0.19 | -19.4 (-12.5- -31.8) | 0.029 | 0.009 |
| 16.Apical lateral | -16.9 (-8.3- -24.3) | -17.6 (-10.2- -22.6) | 0.93 | -21.6 (14.4- -29.5) | 0.013 | 0.004 |
| 17. Apex | -17.5 (-7.2- -22.2) | -21 (-5.8- -26) | 0.47 | -23 (-9.8- -26) | 0.016 | 0.001 |

Strain values are defined as percentage shortening (%). P ^(1)^: Baseline vs. 6 months follow up , P ^(2):^ baseline vs. one year follow up, P ^(3)^: 6 months vs. one year follow up. Values are expressed as median and range.
